# Effects of Nutrient and Water Supply During Fruit Development on Metabolite Composition in Tomato Fruits (*Solanum lycopersicum* L.) Grown in Magnesium Excess Soils

**DOI:** 10.3389/fpls.2020.562399

**Published:** 2020-09-25

**Authors:** Yangmin X. Kim, Min Cheol Kwon, Seulbi Lee, Eun Sung Jung, Choong Hwan Lee, Jwakyung Sung

**Affiliations:** ^1^National Institute of Agricultural Sciences, Rural Development Administration, Wanju, South Korea; ^2^Department of Bioscience and Biotechnology, Konkuk University, Seoul, South Korea; ^3^Department of Systems Biotechnology, Konkuk University, Seoul, South Korea; ^4^Research Institute for Bioactive-Metabolome Network, Konkuk University, Seoul, South Korea; ^5^Department of Crop Science, College of Agriculture, Life and Environment Sciences, Chungbuk National University, Cheongju, South Korea

**Keywords:** water, tomato fruit quality, mineral nutrient, metabolite profiling, lycopene

## Abstract

Tomato cultivation in the greenhouse or field may experience high surplus salts, including magnesium (Mg^2+^), which may result in differences in the growth and metabolite composition of fruits. This study hypothesized that decreasing the supply of nutrients and/or water would enhance tomato fruit quality in soils with excess Mg^2+^ that are frequently encountered in the field and aimed to find better supply conditions. For tomato plants cultivated in plastic pots using a plastic film house soil, the fertilizer supply varied in either the nitrogen (N) or potassium (K) concentration, which were either 0.1 (lowest) or 0.75 times (lower) than the standard fertilizer concentrations. Water was supplied either at 30 (sufficient) or 80 kPa (limited) of the soil water potential. Lycopene content on a dry-weight basis (mg/kg) was enhanced by the combination of lowest N supply and sufficient water supply. However, this enhancement was not occurred by the combination of the lowest N supply and limited water supply. Sugars and organic acids were decreased by limiting the water supply. Therefore, we carefully suggest that an adjustment of nitrogen with sufficient watering could be one of strategies to enhance fruit quality in excess Mg^2+^ soils.

## Introduction

There is worldwide concern about soil salinization reaching 20% of irrigated land, which would cause a reduction in crop growth (Adams et al., 2019). The greenhouse cultivation of tomatoes (*Solanum lycopersicum* L.), including in plastic film houses, involves the application of an extensive amount of fertilizers and produces an accumulation of surplus salts in soil (e.g., Mg^2+^, Ca^2+^, Na^+^, SO_4_^2-^, and Cl^-^). Those surplus salts hinder water transport from soil to roots by producing negative osmotic potential in the soil and by changing root anatomy and root hydraulic conductivity (Adams et al., 2019). Saline drainage water containing not only high Na^+^ but also high Mg^2+^ has been used to cultivate tomatoes in the field (Mitchell et al., 1991). Recently, the effects of Mg oversupply on the metabolite content of tomato plants and fruits were extensively investigated by Kwon et al. and they reported poor fruit quality under excess Mg conditions (Kwon et al., 2019).

To improve fruit quality with enhanced nutrients and functional metabolites, many studies have applied minimal nutrients and irrigation to horticultural plants (Stefanelli et al., 2010). Developing a strategy to apply minimal nutrients and water in agriculture is appropriate considering the current context, e.g., increasing demand for an environmentally friendly strategy and the negative attitude toward genetically modified food (Liu et al., 2015; Carillo et al., 2020). Especially for tomato fruits, optimal nitrogen (N) and potassium (K) supplies in hydroponic tomato cultures were reported to be needed for the best lycopene content that is an antioxidant and a representative functional metabolite of tomato (Simonne et al., 2007; Almeselmani et al., 2010). Either the lowest or the medium level of N supply among the varying applications of N produced best lycopene content in tomato studies both in hydroponics and field studies (Aziz, 1968; Simonne et al., 2007; Kuscu et al., 2014). For K supply, there was a study showing that a medium level of K supply among the varying applications of K produced the best lycopene content of tomatoes in hydroponic cultures (Simonne et al., 2007), although the general trend of K was that increasing K concentration increased lycopene production (Dumas et al., 2003). Deficit irrigation improved the tomato fruit quality by increasing fruit soluble solid levels and concentrations of hexoses, citric acid, and potassium (Mitchell et al., 1991). The enhancement of the lycopene content of tomatoes has been reported under restricted irrigation conditions (Stefanelli et al., 2010; Wang and Xing, 2017). The combination of reduced water supply and high NPK fertilizer concentrations enhanced tomato fruit quality by increasing lycopene, organic acid, and soluble sugar content (Wang and Xing, 2017). The combined effects of nitrogen fertilization and water have been investigated for soluble sugar, vitamin C, and free amino acid contents, not only in tomato fruits but also in cucumber, and it was demonstrated that fruit quality increased under low irrigation conditions and a medium level of N application (Zhang et al., 2011). Therefore, controlling the nutrient and water supply is applicable for the purpose of enhancing fruit quality.

We postulated that minimal nutrient and water supplies may improve tomato quality in soils with surplus salt, which are frequently encountered in the field. Our aim in this study was to provide guidelines to improve tomato fruit quality by nutrient and water regulation in the case of excess Mg^2+^ soils. We hypothesized that minimal nutrient and water use may work in excess Mg^2+^ soil. In order to achieve our goal, the effects of a low nutrient level (N or K) compared to the standard level and/or a reduction in the water supply compared to sufficient water supply were analyzed in the case of excess Mg^2+^ soils. The scope of our study did not include comparing the case of Mg^2+^ excess soils and that of Mg^2+^ non-excess soils. The standard condition was the recommended nutrient supply for tomato cultivation in greenhouses (NIAS, 2017). The lower nutrient level was 0.75 times of the standard (N0.75, K0.75) and the lowest nutrient level was 0.1 times of the standard (N0.1, K0.1). We investigated the resulting fruits’ lycopene content and primary metabolites, including sugars and organic acids, which affect tomato taste. The primary metabolites were measured using a non-targeted metabolomics using a comprehensive approach to evaluate different metabolomes under a specific set of conditions (Son et al., 2016). We have previously applied the method to investigate metabolic changes in various crops, including tomato plants, in response to varying environmental factors, such as light or mineral nutrient supply (Jung et al., 2013; Sung et al., 2015; Kim et al., 2018; Sung et al., 2018).

## Materials and Methods

### Plant Materials, Soil Chemical Properties, and Variations in Nutrient and Water Supply

Three-month-old tomato seedlings (*Solanum lycopersicum* L., ‘Super Dotaerang’) were transplanted into plastic pots (15 L) containing 11 kg of soil on April 5, 2018 and were grown for 16 weeks at daily temperatures between 15–35°C in a greenhouse in the National Institute of Agricultural Sciences, Rural Development Administration, in Jeonju, Korea. Even though the temperature in the greenhouse fluctuated throughout the experimental period, the heating and ventilating systems were operated to avoid extreme temperature conditions (critical lowest and highest temperatures for tomato cultivation). The relative humidity in the greenhouse was not controlled, and natural light penetrating into the greenhouse was the only source of light. The fertilizer supply was in accordance with the recommendations for tomato cultivation in greenhouse soil (NIAS, 2017). For experimental group receiving the standard nutrient supply, the initial fertilizer containing N, P, K, and livestock manure-based compost was supplied at the recommended concentration and the initial fertilizer containing Mg was oversupplied to produce a high level of soil exchangeable Mg^2+^ (over the optimal level, 2.0 cmol kg^-1^ (**Table S1**). Two split applications of N, K, and Mg fertilizer were conducted by top-dressing (**Table S2**). To see the effects of low nitrogen and potassium supplies, the initial and additional fertilizer supplies varied either the N or K concentration by either 0.1 or 0.75 times the standard (N0.1, N0.75, K0.1, K0.75). The soils contained a high concentration of exchangeable Mg^2+^ (3.3–9.0 cmol kg^-1^) after the final harvest. The soil pH was monitored during cultivation and the lowered pH of the soil was amended by applying saturated Ca(OH)_2_ solution. To analyze water supply effects, variations in water supply started from fruit setting; water was supplied either at 30 kPa for the sufficient supply or 80 kPa of soil water potential for the limited supply by reading tensiometers (Soilmoisture Equipment Corp., Santa Babara, USA). The tensiometer’s gypsum block, which senses the water potential, was placed at depth of the 0.07–0.13 m below the soil surface. There were 10 nutrient x water supply conditions. To improve the fruit setting, fully expanded flowers were treated with hormones (gibberellin and 4-chlorophenoxyacetic acid). Ripened fruits from 40 plants were harvested at similar ripening stages at 10:00 to avoid the diurnal changes in metabolites (four plants for each nutrient x water supply condition). One to seven ripened tomato fruits from each plant were harvested from 28 June to 24 July.

### Measurement of Physiological Parameters

Chlorophyll content was measured at the final harvest that was 16 weeks after the mineral nutrient treatments. A chlorophyll meter (SPAD-502Plus, Konica Minolta Sensing, Osaka, Japan) measured the SPAD values for the leaves closest to the tomato fruits (9^th^–10^th^ nodes from the bottom), which were used as an indicator of plant performance rather than as a highly accurate measure of chlorophyll content.

### Sample Preparation and Gas Chromatography-Time of Flight-Mass Spectrometry (GC-TOF-MS) Analysis

Lyophilized fruit samples were ground to powder using a mortar and pestle and were stored at −80°C. For primary metabolite extraction, 100 mg of sample was mixed with 1 ml of 80% (v/v) methanol and sonicated for 10 min. After the samples were homogenized for 10 min at 30 Hz/s by a mixer mill (Retsch GmbH & Co., Haan, Germany), the sample was centrifuged for 10 min at 27,237 × g and 4°C. The supernatants were filtered *via* a 0.2-μm polytetrafluoroethylene filter and were concentrated using a speed vacuum (Modulspin 31, Biotron, Seoul, Korea).

The dried extracts were derivatized through oximation and silylation reactions prior to GC-TOF-MS analysis. The oximation was performed by adding 50 μl of methoxyamine hydrochloride in pyridine (20 mg/ml) to the dried samples and the mixture was incubated for 90 min at 30°C. Subsequent silylation was performed by adding 50 μl of N-methyl-N-(trimethylsilyl)-trifluoroacetamide (MSTFA) to the mixture and incubating the mixture for 30 min at 37°C. A GC-TOF-MS analysis was performed using an Agilent 7890A GC system (Palo Alto, CA, USA) coupled with an Agilent 7693 auto-sampler and a TOF Pegasus III mass spectrometer (LECO, St. Joseph, MI, USA). The derivatized samples were injected with 1 μl with a split ratio of 1:5. For separation, an Rtx-5MS column (i.d. 30 m × 0.25 mm; 0.25 μm particle size; Restek Corp., Bellefonte, PA, USA) was used alongside a helium carrier gas with a flow rate of 1.5 ml/min. The injector and transfer line temperatures were 250 and 240°C, respectively. The oven temperature of the GC was programmed as follows: 75°C for 2 min, increased to 300°C at a rate of 15°C/min, and kept at 300°C for 3 min. Electron ionization was in EI mode at −70 eV, with a scan range of 45–1,000 mass to charge ratio (m/z).

### Lycopene Analysis

For lycopene extraction, each powdered sample (200 mg) was extracted with 3 ml of chloroform/DCM (2:1, v/v) using a twist shaker (Biofree, Seoul, Korea) at 60 rpm for 20 min and then 1 ml of 1M sodium chloride solution was added. Subsequently, the extracts were centrifuged at 5,000 rpm for 10 min at 4°C, and the organic phases were filtered using Millex^®^ GP 0.22 μm filters. The filtered organic phase was completely dried using a speed-vacuum concentrator (Biotron, Seoul, Korea). The dried samples were reconstituted with MeOH/MTBE (tert-Butyl methyl ether) (3:2, v/v) to a final concentration of 10 mg/ml. Lycopene content was analyzed by a UHPLC-DAD system. The UHPLC-DAD system consisted of a Dionex UltiMate 3000 RS Pump, a RS Autosampler, a RS Column Compartment, and a RS Diode Array Detector (Dionex Corporation, Sunnyvale, USA). Chromatographic separation was performed on a YMC carotenoid C30 column (250mm x 4.6mm x 5 μm particle size; YMC, Wilmington, NC) and the injection volume was 10 μl. The flow rate was 0.3 ml/min. The mobile phase consisted of 95% aqueous methanol (A) and MTBE (B). The gradient parameters were set as follows: 20% solvent B was maintained initially for 3 min, followed by a linear increase to 100% solvent B over 25 min and solvent B was then sustained at 100% for 3 min, with a gradual decrease to 20% solvent B over 6 min. The total run time was 37 min and sample absorbance was measured at 200–600 nm. Lycopene was identified based on its retention time and absorbance spectra, which were compared to the retention times of the lycopene standards.

### Data Processing and Multivariate Statistical Analysis

Analyses were conducted of at least 4 biological replicates. The GC-TOF-MS raw data were converted to NetCDF (*.cdf) using the LECO Chroma TOF software (version 4.44). Converted CDF data were preprocessed with the MetAlign software package (http://www.metalign.nl) for peak detection, retention time correction, and alignment. The resulting data were exported to an Excel file. The multivariate statistical analyses, including partial least square-discriminant analysis (PLS-DA) score plot and loading plot, were performed by SIMCA-P+ 12.0 software. Variable importance in the projection (VIP) value was applied to select the discriminant variables among experimental groups. Selected metabolites were tentatively identified by comparisons with various data, including mass fragment patterns, retention times, and mass spectrums of data for standard compounds under the same conditions from published papers and commercial databases, such as the National Institutes of Standards and Technology (NIST) Library (version 2.0, 2011, FairCom, Gaithersburg, MD, USA), and Wiley 8, BioCyc Database Collection (https://biocyc.org/). Significant differences (p < 0.05) were tested by a one-way ANOVA using Statistica (version 7.0, StatSoft Inc., Tulsa, OK, USA).

## Results

### Tomato Plant and Fruit Productions

The leaf chlorophyll content (SPAD value) and fruit yield were measured to compare the effects of mineral nutrient and water supply on shoot growth and fruit production of tomato plants (**Table 1**). Leaf SPAD values generally decreased, although not statistically significantly, as N or K supply was decreased under sufficient water supply. The nutrient supply of N0.75 and N0.1 induced symptoms of yellowish leaves, and the K0.75 and K0.1 supply caused brown dots in the leaves (data not shown). Leaf SPAD values were higher under limited water supply conditions than under sufficient irrigation, and it is speculated that plant growth was slowed by the water limitation, resulting in higher SPAD values. The symptoms in the leaves under N0.75, N0.1, K0.75, and K0.1 supplies were suppressed under limited water supply conditions compared to sufficient water, and this indicates that leaves experienced less nutrient deficiency under limited water than sufficient water condition (see *Discussion*). The leaf SPAD values of N0.1-SW were significantly lower than those of SN-LW and K0.1-LW. The fruit yields under N0.75 and N0.1 applications were similar to the plants receiving the standard nutrient supply; however, plants receiving K0.75 and K0.1 treatments had improved fruit yields compared to the standard. Under all nutrient conditions, the limited water supply reduced fruit production. Nevertheless, none of the detected differences in fruit yield were significant.

**Table 1:** Leaf chlorophyll content (SPAD) and fruit yield per plant cultivated under varied nutrient and water conditions.

Nutrient Supply	Water supply	Abbreviated name	Leaf SPAD at the final harvest	Fruit yield (g/plant)
Standard	Sufficient	SN-SW	42.5 ± 4.7^ab*^	261^ns^
	Limited	SN-LW	44.1 ± 6.8^a^	131
N0.75	Sufficient	N0.75-SW	34.8 ± 12.4^ab^	221
	Limited	N0.75-LW	42.7 ± 6.8^ab^	194
N0.1	Sufficient	N0.1-SW	25.1 ± 6.1^b^	249
	Limited	N0.1-LW	29.5 ± 7.0^ab^	149
K0.75	Sufficient	K0.75-SW	44.8 ± 1.2^a^	272
	Limited	K0.75-LW	39.4 ± 13.1^ab^	146
K0.1	Sufficient	K0.1-SW	33.5 ± 11.6^ab^	415
	Limited	K0.1-LW	45.3 ± 1.3^a^	382

Mean values ± SD of four biological replicates.

^*^Different alphabetical letters in a column indicate significant differences, as determined by ANOVA followed by Tukey’s test (p < 0.05). Ns: no significant differences in a column without letters. SN, standard nutrient; SW, sufficient water; LW, limited water.

### Lycopene Content of Tomato Fruits Cultivated Under Different Mineral Nutrient and Water Supplies

**Figure 1:**
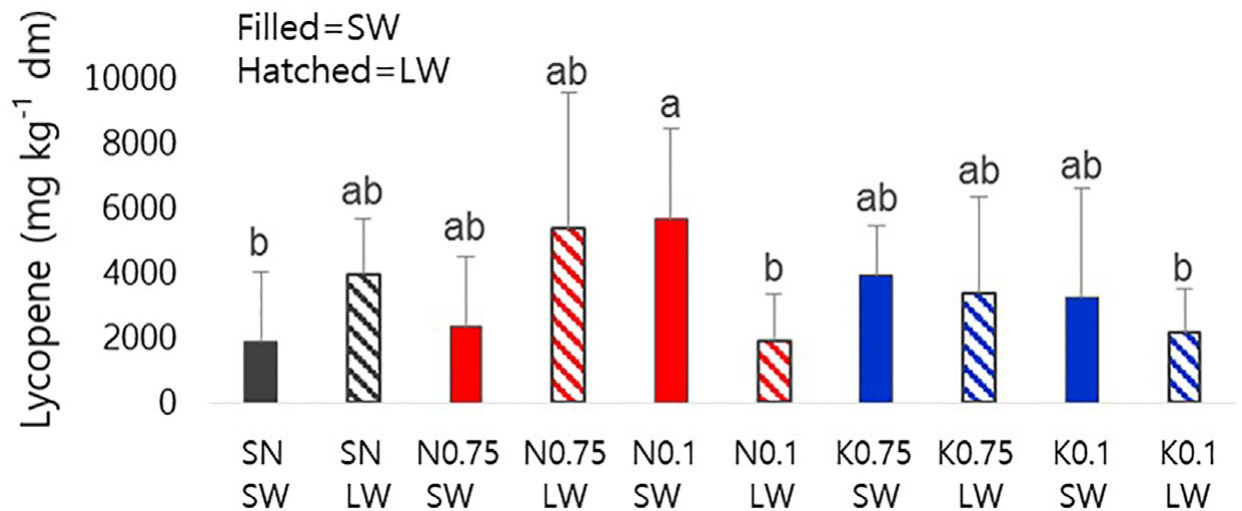
Lycopene contents of tomato fruits on a dry-weight basis influenced by nutrient supply and water supply. The closed bars represent sufficient water and the hatched bars represent limited water. The different alphabetical letters above the bars indicate significant differences, as determined by ANOVA followed by Duncan’s multiple-range test (p < 0.05). SN, standard nutrient; SW, sufficient water; LW, limited water.

We investigated the effect of mineral nutrient and water supply on lycopene content as a representative metabolite for tomato fruit quality (**Figure 1**). In N0.1-SW, lycopene content was significantly enhanced compared to the SN-SW, although the standard deviations were rather high (see *Discussion*). In N0.1-LW, the lycopene-increasing effects of N0.1 disappeared under a limited water supply. There was no significant difference in the effects of varying concentrations of K.

### Non-Targeted Metabolite Profiling of Tomato Fruits Cultivated Under Different Mineral Nutrient and Water Supplies

**Figure 2:**
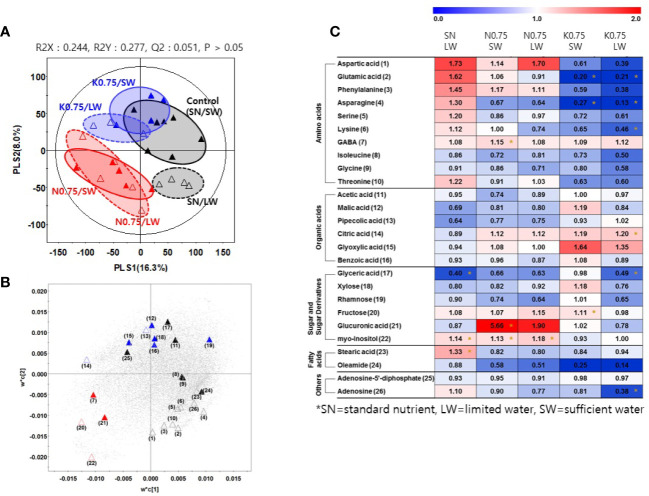
Partial least square discriminant analysis (PLS-DA) score plot **(A)** and a loading plot **(B)** from gas chromatography-time of flight-mass spectrometry (GC-TOF-MS) analysis of tomato fruits grown under the lower nutrient supply (x0.75 of the standard nutrient, SN) and/or limited water (LW) supply compared to the control (SN-SW). ▲: standard nutrient and sufficient water (control, SN-SW), △: standard nutrient and limited water, ▲: x0.75 N and sufficient water, △: x0.75 N and limited water, C▲: x0.75 K and sufficient water, △: x0.75 K and limited water. A heat map **(C)** representing discriminate metabolites among experimental groups with relative metabolite levels. The relative content in the heat map represents fold-changes normalized to the average value of each compound in the control group (SN-SW). The content of each compound is on a dry-weight basis. The number of each metabolite shown in **(B)** is identical to the number in heatmap **(C)**. *: Significantly discriminant metabolites from the control (SN-SW) evaluated by t-test (p < 0.05). SN, standard nutrient; SW, sufficient water; LW, limited water.

**Figure 3:**
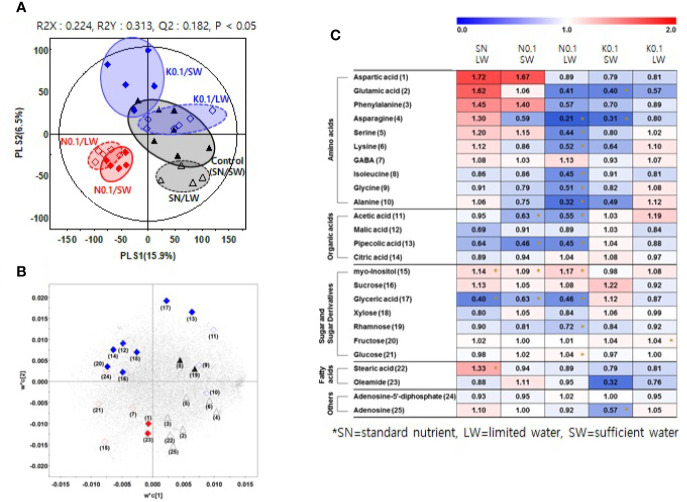
Partial least square discriminant analysis (PLS-DA) score plot **(A)** and loading plot **(B)** from GC-TOF-MS data for tomato fruits grown under the lowest nutrient supply (x0.1 of the standard nutrient, SN) and/or the limited water (LW) supply compared to the control (SN-SW). ▲: standard nutrient and sufficient water (control, SN-SW), △: standard nutrient and limited water, ♦: x0.1 N and sufficient water, ◊: x0.1 N and limited water, ♦: x0.1 K and sufficient water, ◊: x0.1 K and limited water. A heat map **(C)** representing discriminate metabolites among experimental groups with relative metabolite levels. The relative content in the heat map represents fold-changes normalized to the average value of each compound in the control group (SN-SW). The content of each compound is on a dry-weight basis. The number of each metabolite shown in **(B)** is identical number in heatmap **(C)**. *Significantly discriminant metabolites from the control (SN-SW) evaluated by t- test (p < 0.05). SN, standard nutrient; SW, sufficient water; LW: limited water.

We investigated the effects of nutrient and water supply on tomato primary metabolites, including sugars and organic acids (**Table S3**). To investigate the effect of the nutrient and water supply conditions on primary metabolites in tomato fruits, we performed a partial least square discriminant analysis (PLS-DA) for each of the two nutrient levels applied to plants, 0.75 and 0.1 (**Figures 2A** and **3A**, respectively). The clustering patterns of the two PLS-DA results were similar. In **Figures 2**, **3**, the SN and varying the N or K supply (N 0.75, N0.1, K0.75, and K0.1) groups were clearly separated from each other, with PLS1 (16.3%) and PLS1 (15.9%), respectively. In addition, the SW and LW groups were clearly separated from each other, with PLS2 (8.0%) and PLS2 (6.5%), respectively. The limited water groups with mineral applications of N0.75 and K0.75 were not clearly discriminated from the sufficient water supply groups. However, the limited water groups with mineral applications of N0.1 and K0.1 were clearly discriminated from the groups with sufficient water supply. These results indicate that the amount of nutrients supplied to plants affects the primary metabolites of tomato fruits more than irrigation conditions.

Based on the PLS-DA models, we selected significantly discriminant metabolites using variable importance in projection (VIP) values of >0.7. The same VIP values were selected by Azam et al. (2020) and Ibrahim et al. (2020) in metabolomic studies. A total of 26 and 25 significantly discriminant metabolites were selected and identified for the two nutrient levels of 0.75 and 0.1 times the standard application concentrations, respectively (**Figures 2B**, **3B**). The relative primary metabolite levels of tomato fruits compared to the control, SN-SW, are shown in heat map (**Figures 2C**, **3C**). The effect of water limitation under standard nutrient conditions was analyzed by comparing SN-LW to SN-SW. Water limitation under standard nutrient conditions increased the amount of some amino acids but decreased the levels of most of the carbohydrates and organic acids present in the fruit (**Figures 2C**, **3C**). The effect of water limitation under lower nutrient supply was also analyzed in pairs, namely N0.75-SW/N0.75-LW, K0.75-SW/K0.75-LW, N0.1-SW/N0.1-LW, and K0.1-SW/K0.1-LW. Water limitation under the lowest N condition resulted in a drastic decrease in the amount of amino acids (**Figure 3C**). A similar effect of water limitation was detectable with K0.75-LW but not K0.1-LW (**Figure 2C**). Interestingly, changes in metabolite content compared to the control, SN-SW, were greater under the x0.75 nutrient condition than under the x0.1 nutrient condition. The amounts of most of the compounds were lowered by reduction of the N and K supply compared to SN-SW. However, there were some exceptions. For example, the level of aspartic acid, phenylalanine, and glucuronic acid were highly increased in N0.75 samples, while in N0.1 samples, only aspartic acid and phenylalanine were present in higher amounts than in the control. Lowering the K supply had a negative effect, mainly on amino acid content, and especially on asparagine levels, which were lowered in both K0.75 and K0.1 samples. In contrast, the glyoxylic acid concentration was highly elevated in K0.75, but not in K0.1 fruits.

## Discussion

Lycopene is one of the most potent antioxidants produced in tomato fruits. Although there were considerable fluctuations of lycopene content due to the extreme environmental conditions in the growing season, we found a significant increase in lycopene content of tomato fruits grown under the lowest N supply with standard irrigation. Simonne et al. (2007) showed that optimal N supply is needed to reach high lycopene concentrations in tomato grown in hydroponic culture. Our results suggest that in soil with excess Mg^2+^, the optimal N supply for lycopene production is x0.1 of the standard amount used.

The analysis of primary metabolites of tomato fruits by non-targeted metabolomics approaches revealed the changes caused by a limited water supply. The limited water supply decreased most of the carbohydrates and organic acids on a dry-weight basis. Therefore, limiting water was not recommended to improve tomato fruit quality in Mg^2+^ excess soil. There were reports that water deficit increased the acidity of tomato fruits (Rudich et al., 1977; 1985 experiment of Mitchell et al., 1991), however, acid levels stayed similar in the experiment of 1986 of Mitchell et al. (1991). In the latter study, hexose increased on a fresh-weight basis in 1985 but hexose stayed similar in 1986 when fruit water content was not changed (Mitchell et al., 1991). They concluded that the production of solutes such as hexoses and organic acids was not up-regulated but that fruit water content decreased, resulting in the increase in metabolite concentration on a fresh-weight basis.

Lower nutrient supply caused a reduction in most of the primary metabolite content of fruits. The observation of reduced concentrations of organic acids under the lower and lowest N conditions in this study is in line with the literature in which low N was found to decrease organic acid concentrations (Bénard et al., 2009; Kuscu et al., 2014; De Pascale et al., 2016; Wang and Xing, 2017). No significant changes in carbohydrates in tomato fruits under the lower and lowest K supplies were detected, which seems to contrast common knowledge about the effects of K on fruits. K is known to be important to high sink activity, as K deficiency causes sugar accumulation in the source, such as shoots, and reduces sugar transport to sinks, such as roots or fruits (Cakmak et al., 1994; Kanai et al., 2011; Hafsi et al., 2014; Kim et al., 2018). The K deficiency effect may not be significant in the current study, and this could be related to the fact that high concentrations of Mg^2+^ in soil are not known to reduce K^+^ uptake by plants (Gransee and Führs, 2013). In contrast to Mg stress, for tomatoes under salt stress from NaCl the uptake and translocation of K was highly hindered when K supply was low (Al-Karaki, 2000).

This study demonstrates that minimizing N supply helped enhance lycopene content in soil with excess Mg^2+^, which was a strategy known to work in soils without Mg^2+^ surplus, so long as irrigation was sufficient. However, combining the lowest N conditions and limited water worsened the lycopene content in excess Mg^2+^ soil. For the best quality of tomato fruits in terms of sugars and organic acids, limiting water was not appropriate in excess Mg^2+^ soil, which was contrary to the conventional strategy for managing fruit production in soils without Mg^2+^ surplus.

## Conclusion

In summary, we examined the effects of nutrient and water supply on metabolic changes in tomato fruits cultivated in soil with excess Mg^2+^ under greenhouse conditions. As the growth, metabolites, and fruit quality of tomato plants under excess Mg^2+^ soil were affected differently than plants in soils without excess Mg^2+^ in Kwon et al. (2019), the effects of nutrient and water were contrary to previous results. Our results suggest that the lowest nitrogen coupled with sufficient water supply condition enhanced the lycopene content of tomato fruits on a dry-weight basis, and that limiting water supply in any nutrient supply condition was not appropriate to enhance sugars and organic acids under greenhouse cultivation when soil had excess Mg^2+^. The current pot experiments in the greenhouse demonstrated the possibility that supplying low levels of nitrogen without limiting water may work in tomato cultivation in the field with overuse of chemical fertilizer, and that this type of strategy needs to be further investigated in the field to set up guidelines for fertilizer supply recommendations.

## Data Availability Statement

All datasets presented in this study are included in the article/**Supplementary Material**.

## Author Contributions

Conceptualization: SL and JS. Validation: SL, EJ, and JS. Investigation: SL, YK, and MK. Resources: SL and JS. Writing—original draft preparation: YK and MK. Writing—review and editing: YK, SL, EJ, CL, and JS. Supervision, CL. Project administration: SL and YK. Funding acquisition: SL.

## Funding

This research was funded by the “Cooperative Research Program for Agriculture Science & Technology Development (Project No. PJ012523, PJ014977)” and by 2020 RDA Fellowship Program of Rural Development Administration, Republic of Korea.

## Conflict of Interest

The authors declare that the research was conducted in the absence of any commercial or financial relationships that could be construed as a potential conflict of interest.
