# Supplementary material for: Effects of Nutrient and Water Supply During Fruit Development on Metabolite Composition in Tomato Fruits (Solanum lycopersicum L.) Grown in Magnesium Excess Soils
Source: Front Plant Sci. 2020 Sep 25;11:562399. doi: 10.3389/fpls.2020.562399 (PMC7545823; doi:10.3389/fpls.2020.562399)
Supplement: Supplementary file 1 [file Table_1.docx]

**Table S1.** Chemical properties of soils after growing tomato plants cultivated under varied nutrient and water conditions. Mean values of three replicates. SN: standard nutrient, SW: sufficient water, LW: limited water.

| Nutrient  supply | Water  suply | Name | pH (1:5) | EC (dS/m) | NO_3_- N (mg/kg) | OM (g/kg) | Av. P_2_O_5_ (mg/kg) | Ex. Cations (cmol_c_^+^/kg) | | | |
| --- | --- | --- | --- | --- | --- | --- | --- | --- | --- | --- | --- |
|  |  |  |  |  |  |  |  | K | Ca | Mg | Na |
| Standard | Sufficient | SN-SW | 5.3 | 3.4 | 14.7 | 32 | 322 | 0.09 | 2.7 | 4.6 | 0.3 |
|  | Limited | SN-LW | 4.9 | 5.7 | 6.2 | 33 | 326 | 0.17 | 2.8 | 6.2 | 0.4 |
| N0.75 | Sufficient | N0.75-SW | 5.3 | 3.8 | 8.5 | 28 | 324 | 0.18 | 2.7 | 4.3 | 0.3 |
|  | Limited | N0.75-LW | 5.1 | 3.4 | 3.2 | 27 | 315 | 0.12 | 2.3 | 3.7 | 0.3 |
| N0.1 | Sufficient | N0.1-SW | 5.3 | 7.0 | 3.7 | 33 | 299 | 0.13 | 2.7 | 9.0 | 0.4 |
|  | Limited | N0.1-LW | 4.9 | 7.3 | 3.7 | 35 | 323 | 0.30 | 2.9 | 7.8 | 0.4 |
| K0.75 | Sufficient | K0.75-SW | 5.3 | 4.8 | 6.2 | 27 | 329 | 0.06 | 2.4 | 5.5 | 0.3 |
|  | Limited | K0.75-LW | 5.1 | 5.9 | 4.6 | 27 | 386 | 0.13 | 2.6 | 7.0 | 0.4 |
| K0.1 | Sufficient | K0.1-SW | 5.9 | 1.9 | 8.7 | 26 | 307 | 0.07 | 2.6 | 3.3 | 0.3 |
|  | Limited | K0.1-LW | 5.2 | 3.7 | 3.9 | 27 | 311 | 0.02 | 2.0 | 4.7 | 0.3 |
