# Supplementary material for: Effects of Nutrient and Water Supply During Fruit Development on Metabolite Composition in Tomato Fruits (Solanum lycopersicum L.) Grown in Magnesium Excess Soils
Source: Front Plant Sci. 2020 Sep 25;11:562399. doi: 10.3389/fpls.2020.562399 (PMC7545823; doi:10.3389/fpls.2020.562399)
Supplement: Supplementary file 2 [file Table_2.docx]

**Table S2.** Fertilizer application for the standard nutrient supply group.

| Fertilizer  application | Time | Fertilization (g/kg soil) | | | |
| --- | --- | --- | --- | --- | --- |
|  |  | N | P | K | Mg |
| Initial supply | Before  transplanting | 0.15 | 0.14 | 0.05 | 0.51 |
| 1^st^ additional supply | 2 months after transplant | 0.01 | 0 | 0.01 | 0.10 |
| 2^nd^ additional supply | 2.5 months after transplant | 0.03 | 0 | 0.03 | 0.30 |

Fertilizer supply was according to the recommendation for tomato cultivation in greenhouse soil.
