# Supplementary material for: Effects of Nutrient and Water Supply During Fruit Development on Metabolite Composition in Tomato Fruits (Solanum lycopersicum L.) Grown in Magnesium Excess Soils
Source: Front Plant Sci. 2020 Sep 25;11:562399. doi: 10.3389/fpls.2020.562399 (PMC7545823; doi:10.3389/fpls.2020.562399)
Supplement: Supplementary file 3 [file Table_3.docx]

**Table S3.** Differential primary metabolites identified by gas chromatography-time of flight-mass spectrometry (GC-TOF-MS) in tomato fruit samples cultivated under varied nutrient supply conditions and water supply conditions.

| **NO.** | **Tentative identification ^a^** | **GC-TOF-MS** | | | | |
| --- | --- | --- | --- | --- | --- | --- |
|  |  | **RT(min) ^b^** | **Identified**  **ion (m/z) ^c^** | **Mass Fragment** | **TMS ^d^** | **ID ^e^** |
| *Amino acids* | | | | |  |  |
| 1 | Aspartic acid | 9.43 | 232 | 73 156 232 100 45 75 | 3 | STD |
| 2 | Glutamic acid | 10.20 | 246 | 73 246 75 128 45 84 | 3 | STD |
| 3 | Phenylalanine | 10.30 | 218 | 73 218 45 192 100 147 | 2 | STD |
| 4 | Asparagine | 10.62 | 116 | 73 116 45 75 132 147 | 3 | STD |
| 5 | Serine | 8.03 | 204 | 73 204 100 45 218 147 | 3 | STD |
| 6 | Lysine | 12.46 | 156 | 156 174 128 86 100 230 | 4 | STD |
| 7 | GABA | 9.50 | 174 | 147 73 86 59 45 304 216 | 3 | STD |
| 8 | Isoleucine | 7.39 | 158 | 73 158 45 100 218 159 | 2 | STD |
| 9 | Glycine | 7.53 | 174 | 174 73 86 45 100 59 | 3 | STD |
| 10 | Threonine | 8.28 | 219 | 73 57 117 45 101 219 | 3 | STD |
| 11 | Alanine | 5.40 | 116 | 116 73 45 75 117 74 | 2 | STD |
| *Organic acids* | |  |  |  |  |  |
| 12 | Acetic acid | 5.10 | 205 | 73 147 45 66 148 59 | 2 | STD |
| 13 | Malic acid | 9.15 | 101 | 73 147 45 75 55 133 | 3 | STD |
| 14 | Pipecolic acid | 9.27 | 186 | 84 75 73 45 158 100 | 1 | NIST |
| 15 | Citric acid | 11.73 | 273 | 147 211 273 183 221 149 | 4 | STD |
| 16 | Glyoxylic acid | 5.51 | 142 | 75 73 45 47 142 56 | 1 | STD |
| 17 | Benzoic acid | 6.93 | 179 | 105 77 179 135 51 45 | 1 | STD |
| *Sugar and Sugar derivatives* | |  |  |  |  |  |
| 18 | Glyceric acid | 7.75 | 102 | 73 147 189 45 102 103 | 3 | NIST |
| 19 | Xylose | 10.60 | 307 | 73 103 217 147 74 307 | 4 | STD |
| 20 | Rhamnose | 11.11 | 117 | 73 117 75 131 70 118 | 4 | STD |
| 21 | Fructose | 12.28 | 307 | 147 217 103 133 307 117 | 5 | STD |
| 22 | Glucuronic acid | 12.67 | 333 | 73 160 147 333 45 143 | 3 | STD |
| 23 | Myo-Insitol | 13.59 | 265 | 73 147 217 191 129 133 | 6 | STD |
| 24 | Glucose | 12.43 | 160 | 160 147 103 117 133 | 5 | STD |
| 25 | Sucrose | 16.66 | 271 | 73 103 217 362 147 74 | 8 | STD |
| *Fatty acids* | |  |  |  |  |  |
| 26 | Stearic acid | 14.28 | 117 | 117 75 73 129 132 55 | 1 | STD |
| 27 | Oleamide | 15.27 | 131 | 75 73 131 144 116 128 | 1 | STD |
| *Others* | |  |  |  |  |  |
| 28 | Adenosine-5`-diphosphate | 7.18 | 299 | 73 299 45 133 207 193 | 3 | STD |
| 29 | Adenosine | 16.54 | 236 | 73 230 236 103 45 217 | 4 | STD |

^a^ Metabolites selected by a VIP value of > 0.7 based on each PLS-DA model (Figs. 2a, 3a); ^b^ Retention time; ^c^ The selected ion is the m/z value for identification; ^d^ TMS : trimethylsilyl groups; ^e^ Identification. MS, mass spectrum was confirmed with the National Institutes of Standards and Technology (NIST) database and in-house libraries; STD, mass spectrum was consistent with that of the standard compounds.
